# Efficacy of using pentoxifylline in patients undergoing breast cancer surgery

**DOI:** 10.3389/fphar.2025.1560805

**Published:** 2025-07-16

**Authors:** Samar A. Dewidar, Noha O. Mansour, Omar Hamdy, Dina A. Elebedy, Moetaza M. Soliman, Mohamed E. E. Shams

**Affiliations:** ^1^ Clinical Pharmacy and Pharmacy Practice Department, Faculty of Pharmacy, Mansoura University, Mansoura, Egypt; ^2^ Surgical Oncology Department, Oncology Center, Mansoura University, Mansoura, Egypt; ^3^ Anasthesia, Surgical Intensive Care and Pain Management Department, Faculty of Medicine, Mansoura University, Mansoura, Egypt

**Keywords:** postoperative, pain, numeric rating scale, RCT, AUC

## Abstract

**Introduction:**

Breast cancer surgery presents several challenges, including postoperative pain, and wound healing complications. Pentoxifylline is a synthetic methylxanthine derivative known for its anti-inflammatory properties and ability to improve microcirculation, decreasing the inflammatory markers as well as restoring the antioxidant status. This study aims to investigate the potential benefits of pentoxifylline in improving pain control and wound healing in patients undergoing mastectomy.

**Methods:**

In a randomized, single-blinded clinical trial, ninety-two breast cancer patients were assigned to receive pentoxifylline or not. The primary outcome was the measurement of postoperative pain level using the Numeric Rating Scale (NRS) at multiple time points within 24 h post-surgery. Secondary outcomes included determining the time till wound healing and the incidence of postoperative complications.

**Results:**

Eighty-eight participants completed this study, 42 patients in the control group while 46 patients in the pentoxifylline group. Patients receiving pentoxifylline demonstrated a significant decrease in NRS scores as compared to the control group (median (IQR) of total area under the curve (AUC) over 24 h were 90 (73.5–102), and 153 (123–168), respectively (*P* < 0.001)), indicating clinically meaningful reductions in pain intensity. Additionally, pentoxifylline-treated patients experienced faster wound healing, reflected by earlier suture removal (mean ± SD: 15 ± 4.4 days vs. 19.3 ± 6.7 days; respectively (*P*= 0.001)). The incidence of postoperative complications was significantly lower in the pentoxifylline group (2.2%) compared the control group (19%)*, P= 0.01*. Fewer cases of seroma, wound infection, and wound dehiscence were observed in the pentoxifylline group.

**Conclusion:**

Preoperative oral administration of pentoxifylline in patients undergoing breast surgery may reduce postoperative pain and improve recovery in those patients. However, further investigations are imperative to validate these findings.

**Clinical Trial Registration:**

https://clinicaltrials.gov/study/NCT06087237, identifier NCT06087237.

## Graphical Abstract

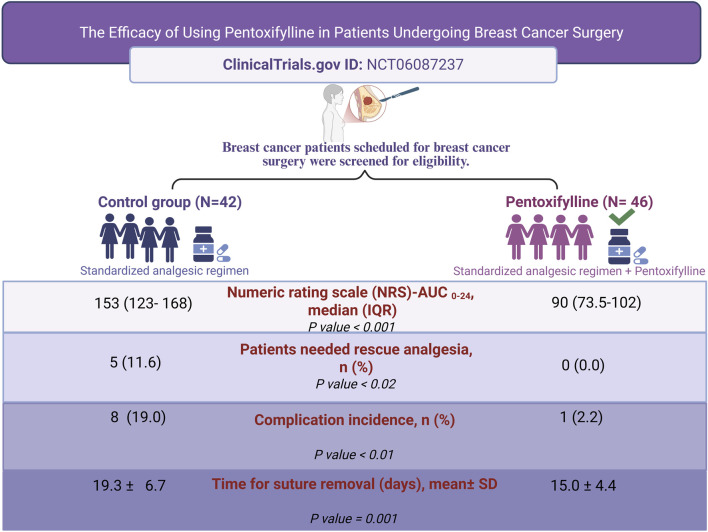

## 1 Introduction

Surgical intervention is a cornerstone of breast cancer management. However, breast cancer surgery presents several challenges, including postoperative pain, wound healing complications, and the potential for other post-surgical complications (National Comprehensive Cancer Network, 2024). These complications negatively impact the patient’s quality of life (Colakoglu et al., 2011).

Many patients experience moderate to severe postoperative pain (Jacobs et al., 2020; Meijuan et al., 2013; Munk et al., 2023). Thus, effective postoperative pain management following breast cancer surgery is crucial. Despite pain intensity typically decreases after surgery, some patients might have persistent postoperative pain for up to 3 months post-surgery, emphasizing the need for effective analgesic strategies (Jacobs et al., 2020; Meijuan et al., 2013; Munk et al., 2023).

Effective pain management is crucial to enhance recovery, reduce complications, and minimize the risk of chronic pain. Recent evidence-based guidelines, such as those from the PROSPECT group, provide a structured approach to perioperative analgesia for breast surgery. These recommendations support the use of scheduled paracetamol and NSAIDs as the core of standardized pain management in the immediate postoperative period after breast cancer surgery, reserving opioids for breakthrough pain (Jacobs et al., 2020). Morphine has always been considered the gold standard analgesic; however, opioids have multiple side effects (Abraham, 2013). Nonetheless, even with this regimen, some patients continue to experience significant pain. Consequently, there is ongoing interest in adjunctive therapies that target additional pain pathways and inflammatory processes (Joshi, 2023). Delayed wound healing is one of the frequent complications after breast cancer surgery (Fife and Carter, 2012; Spira et al., 2018). Wound complications, including seroma, wound dehiscence and infections are significant concerns following oncologic breast surgery. Discovering a therapy that effectively alleviates pain while accelerating patient recovery by enhancing wound healing, and reducing complications is advantageous.

Pentoxifylline is a synthetic methylxanthine derivative with established clinical uses, including treating intermittent claudication. Its mechanism of action involves inhibiting phosphodiesterase, leading to increased levels of cyclic adenosine monophosphate (cAMP) (Salehi et al., 2017). This effect, along with pentoxifylline’s anti-inflammatory properties, improved microcirculation, and modulation of immune responses (Ambrus et al., 1995; Fantin et al., 2006; Szczepanik et al., 2004). These actions suggest potential benefits in managing postoperative pain, enhancing wound healing, and reducing complications.

Recent evidence supports pentoxifylline’s potential in reducing postoperative pain in various surgical settings, such as laparoscopic appendectomy (Nazemi et al., 2021). Pentoxifylline has also demonstrated efficacy in relieving pain associated with conditions such as diabetic nephropathy (Dastgheib et al., 2022), disc hernia (Tarabay et al., 2022), and irritable bowel syndrome (El-Haggar et al., 2022). Pentoxifylline has also shown positive outcomes in various wound healing contexts (Ahmadi and Khalili, 2016) such as colorectal anastomosis (Parra-Membrives et al., 2007; Sümer et al., 2011), post-burn scars (Isaac et al., 2010), radiation-induced injuries (Jacobson et al., 2013; Mimura et al., 2009), and venous ulcers (Jull et al., 2012). Additionally, pentoxifylline has been shown to promote wound healing, as evidenced by studies examining its effects on wound healing following mastectomy and other surgeries (Vorakulpipat et al., 2023).

The associated side effects of pentoxifylline therapy, including indigestion, nausea, vomiting, dizziness, headaches, and angina, are generally mild and well-tolerated, allowing for doses up to 2,500 mg per day (Samlaska and Winfield, 1994; Ward and Clissold, 1987).

Despite promising preclinical and observational findings, the efficacy and safety of pentoxifylline specifically in the context of breast cancer surgery are lacking. This study aimed to investigate the potential benefits of pentoxifylline in improving pain management, and wound healing in patients undergoing mastectomy.

## 2 Methods

### 2.1 Study design

This single-center, parallel, single blinded, randomized controlled study was conducted at the Oncology Center of Mansoura University (OCMU), Egypt. The protocol was approved by the institutional review board at the Faculty of Pharmacy, Mansoura University (code 2023–147). The study protocol was registered at clinicaltrials.gov (NCT06087237) prior to the patients’ enrollment. The study was performed according to the Declaration of Helsinki. All participants provided written informed consent prior to study participation.

### 2.2 Participants

Breast cancer patients were screened for eligibility if they were scheduled for breast cancer surgery. The study included adult female patients (aged 18–65 years) who had American Society of Anesthesiologists (ASA) physical status I and II. The exclusion criteria included patients on treatment regimens of phosphodiesterase inhibitors, antiplatelets or anticoagulants, and those on chronic pain management regimens. Patients allergic to phosphodiesterase inhibitors, those with a history of recent bleeding events, active peptic ulcer or psychological problems were excluded from this study.

### 2.3 Randomization, blinding and study interventions

Participants were randomized in a 1:1 ratio using a computer-generated permuted-block randomization with a fixed block size of six. Within each block, assignment orders were randomly permuted, and the sequence was finalized using a published random-number table. Patients were either enrolled in the pentoxifylline group or the control group. Patients in the pentoxifylline group received 800 mg of pentoxifylline tablets (two tablets/400 mg each) 2 h before the operation and continued treatment with one tablet administered every 8 h postoperatively during hospitalization, which was maintained for 4 weeks post-discharge. Patients in the control group did not receive placebo tablets. A standardized pain management protocol was initiated for all participants in both groups upon arrival in the Post-Anesthesia Care Unit (PACU), consisting of intravenous acetaminophen (1 g) every 8 h and ketorolac 30 mg IV every 12 h during hospitalization. Patients were only allowed to receive a rescue analgesic regimen comprised of 2 mL nalbuphine (20 mg/mL) if they had severe pain and requested additional analgesia. Use of rescue analgesia was recorded during hospitalization. Patients and investigators directly involved in patient care were aware of treatment assignment; however, outcome assessors, who did not participate in any clinical care, remained unaware of group allocation.

The operation was conducted under general anesthesia. Induction of anesthesia began with 2 μg/kg of fentanyl and 2 mg/kg of propofol until the loss of response to verbal commands was achieved. Atracurium (0.5 mg/kg IV) was administered to facilitate tracheal intubation and maintain muscle relaxation as required. Mechanical ventilation was provided using a circle system with a 50% oxygen and air mixture to sustain end-tidal carbon dioxide levels within the range of 35–45 mmHg. Upon completion of the surgical procedure, muscle relaxation was reversed using atropine (0.01 mg/kg) and neostigmine (0.04 mg/kg). No additional pain medication was administered during the procedure, and the duration of each surgery was documented. Once conscious and responsive to verbal commands, patients were transferred to the PACU.

Post-discharge, adherence to the study intervention in the pentoxifylline group was confirmed by pill count at every visit during the follow-up period until the end of the study. Patients with less than 95% adherence to the treatment regimen were excluded from the analysis.

### 2.4 Study endpoints

#### 2.4.1 Primary efficacy endpoint

The primary outcome of this study was to compare the severity of postoperative pain levels between the two groups. The severity of pain was assessed by the Numeric Rating Scale (NRS) using consecutive assessments over the course of 24 h after the surgery (0, 6, 12, 18, 24 h post-operation). Prior to surgery, patients were instructed to use the NRS to quantify their pain, where 0 represented no pain and 10 denoted the most severe pain. Postoperatively, pain assessments were conducted every 6 h, starting immediately in the PACU and before initiation of the analgesic regimen. A physician, who was blinded to study interventions, independently recorded the NRS score of the patient’s pain level. The area under the curve (AUC) of NRS scores was calculated using the trapezoidal rule (Abdelaziz et al., 2021; Emara et al., 2023), resulting in an overall NRS-AUC score for each patient (Lang-Illievich et al., 2020; Schwartz et al., 2024). A reduction of more than 1.41 points in the patient assigned NRS was considered to be clinically important (Kendrick and Strout, 2004).

#### 2.4.2 Secondary efficacy endpoints

Rescue analgesia requirement was assessed by the proportion of patients requiring rescue opioid analgesia during hospitalization, and the cumulative doses were compared between the study arms.

Time to wound healing was assessed by comparing the average time to wound closure in both groups, as reflected by the time of suture removal after closure of the approximate wound edge during weekly follow-up visits for 4 weeks post-discharge.

Incidence of postoperative complications was evaluated by the incidence of short-term postoperative complications in each group, which encompassed infection of the surgical site, seroma (fluid accumulation at the surgical site necessitating drainage), and wound dehiscence (opening of the surgical wound, denoted as a wound healing issue). These complications were evaluated based on consultants’ notes (blinded to intervention) during weekly follow-up visits postoperatively.

#### 2.4.3 Safety endpoints

Throughout the study, the prevalence of the most frequent pentoxifylline adverse effects was noted. The adverse effects that have been reported include headache, nausea, vomiting, diarrhea, bloating, abdominal discomfort, and dizziness (Annamaraju and Baradhi, 2024).

### 2.5 Sample size calculation

The required sample size was calculated using G*Power software version 3.1.0. According to previous data (Nazemi et al., 2021), a sample size of 80 individuals was required to provide 80% power with a 2-sided test, α level of 0.05 and an effect size of 0.63 for the pain intensity score after surgery between the two groups. Accounting for an attrition rate of 15%, a total sample size of 92 patients were randomized in the present study.

### 2.6 Statistical analysis

Statistical analysis was performed using Jamovi statistical software version 2.6. Per-protocol analysis was conducted. Data of categorical variables were summarized as frequencies and percentages. Pearson’s chi-square tests were used to compare the categorical variables in the two groups. Data of the quantitative variables were represented as mean and standard deviation (SD) if normally distributed or median and interquartile ranges (IQR) if not normally distributed. To test data normality, we used the Shapiro-Wilk test. The Student’s t-test was used to compare the difference in normally distributed data between the two groups, while the Mann-Whitney U test was utilized for quantitative data that did not follow the parametric assumption. Kaplan-Meier survival analysis was used to study time to suture removal, and the two groups were compared by the log-rank test. *P-value* < 0.05 was considered statistically significant.

## 3 Results

**FIGURE 1:**
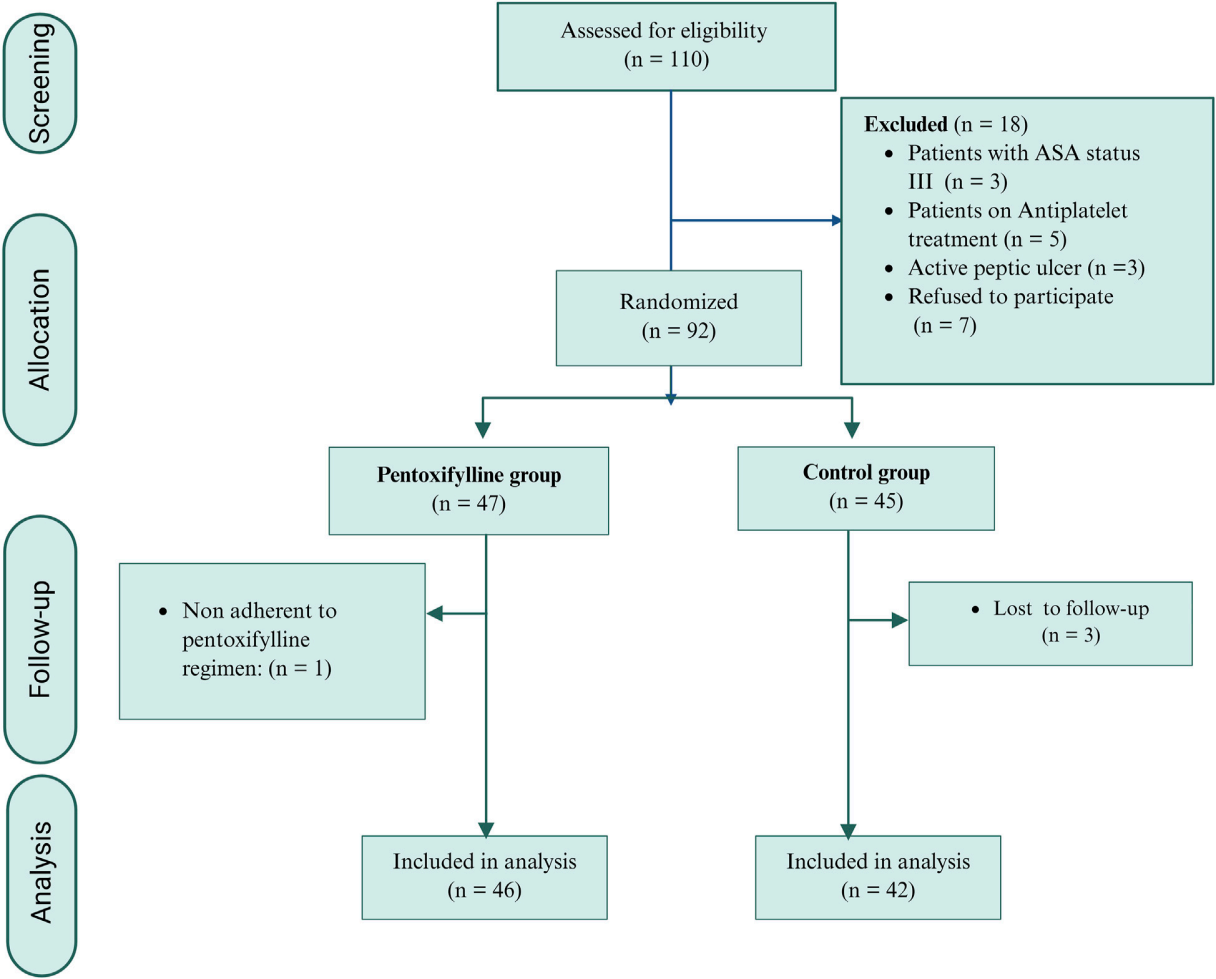
Patient flow chart.

Between November 2023 and June 2024, one hundred and ten breast cancer patients intended for breast surgery were screened for eligibility criteria. Ninety-two patients were enrolled in the study. As shown in Figure 1, three patients in the control group were lost to follow-up, and one patient in the pentoxifylline group was excluded due to nonadherence to the study protocol. These cases were excluded from the per-protocol analysis. Consequently, eighty-eight patients were included in the final analysis: 42 in the control group and 46 in the pentoxifylline group.

At baseline, the two groups were comparable regarding age, body mass index (BMI), body surface area (BSA), coagulation profile, blood glucose level, comorbid conditions, ASA status, type of breast surgery, lymph node removal type and duration of surgery (Table 1).

**TABLE 1:** Baseline patient demographics and surgery characteristics.

Parameter	Control group (n = 42)	Pentoxifylline group (n = 46)	p-value
Age (year), mean ± SD	52.0 ± 11.6	52.6 ± 10.3	0.82^a^
BMI (kg/m^2^), mean ± SD	34.5 ± 6.9	34.3 ± 6.6	0.88^a^
BSA (m^2^), mean ± SD	1.9 ± 0.2	1.9 ± 0.2	0.68^a^
PT (seconds), median (IQR)	12.0 (0)	12.0 (0)	0.67^b^
INR, median (IQR)	1.0 (0.01)	1.0 (0.05)	0.41^b^
Blood glucose (mg/dL), median (IQR)	107.5 (25.8)	107.5 (44.5)	0.80^b^
ASA-scale, n (%)			
I II	25 (59.5) 17 (40.5)	32 (69.6) 14 (30.4)	0.33^c^
Comorbidities, n (%)			
None Diabetes Hypertension Hypertension and diabetes Hypertension, diabetes, and CKD Hypertension and ischemic heart disease	25 (59.5) 10 (23.8) 6 (14.3) 1 (2.4) 0 (0.0) 0 (0.0)	32 (69.6) 3 (6.5) 4 (8.7) 4 (8.7) 2 (4.3) 1 (2.2)	0.09^c^
Chronic medications, n (%)			
Bisoprolol Hydrochlorothiazide Enalapril Olmesartan Spironolactone Captopril Insulin Oral hypoglycemics Atorvastatin Chlordiazepoxide + Clidinium bromide Mebeverine Silymarin Levothyroxine	2 (4.7) 4 (9.5) 2 (4.7) 0 (0.0) 0 (0.0) 1 (2.4) 1 (2.4) 9 (21.3) 0 (0.0) 0 (0.0) 0 (0.0) 0 (0.0) 1 (2.4)	5 (10.9) 3 (6.5) 0 (0.0) 2 (4.3) 1 (2.17) 0 (0.0) 2 (4.3) 6 (13.0) 1 (2.17) 1 (2.17) 1 (2.17) 1 (2.17) 2 (4.3)	0.42^c^
Surgery type, n (%)			
Conservative breast surgery Mastectomy Mastectomy and reconstruction	21 (50.0) 17 (40.5) 4 (9.5)	24 (52.2) 20 (43.5) 2 (4.3)	0.63^c^
Lymph node surgery, n (%)			
Sentinel lymph node biopsy Axillary lymph node dissection	17 (40.5) 27 (59.5)	23 (50.0) 23 (50.0)	0.37^c^
Pain level before surgery (NRS), median (IQR)	0 (0)	0 (0)	-
Duration of surgery (minute), median (IQR)	120 (60.0)	120 (52.5)	0.32^b^

The used tests are:

^a^ Student’s t-test.

^b^ Mann-Whitney U.

^c^ Pearson chi square test.

ASA, american society of anesthesiology; BMI, body mass index; BSA, body surface area; CKD, chronic kidney disease; IQR, interquartile range; INR, international normalized ratio; n, number of patients; NRS, numeric rating scale; PT, prothrombin time; SD, standard deviation.

**FIGURE 2:**
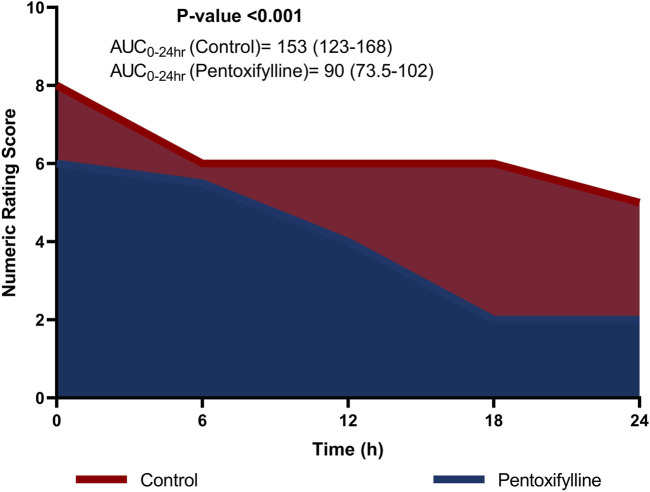
Area graph representing the median (interquartile range) numeric rating scale (NRS) score at the different time intervals in the two groups.

Patients in the pentoxifylline group reported significantly lower NRS score at 0, 6, 12, 18, 24 h post-operation in comparison to those in the control group (*P* < 0.001 at each time point; Table 2). The difference in pain severity between the two groups reached the minimal clinically important difference in pain reduction (≥1.41 points reduction in NRS (Kendrick and Strout, 2004)) at all time points except at 6 h. Additionally, the resultant cumulative AUC was smaller than that in the control group (*P* < 0.001; Figure 2).

**TABLE 2:** Primary efficacy endpoints.

Parameter	Control group (n = 42)	Pentoxifylline group (n = 46)	*P*-value
AUC _0–6 h_	42 (39–48)	30 (27–36)	<0.001^a^
AUC _6–12 h_	36 (30–48)	24 (24–30)	<0.001^a^
AUC _12–18 h_	36 (30–42)	18 (12–24)	<0.001^a^
AUC _18–24 h_	30 (24–36)	12 (9.75–18)	<0.001^a^
AUC Total _(0–24 h)_	153 (123–168)	90 (73.5–102)	<0.001^a^
NRS 0 h	8 (7–9)	6 (4–8)	<0.001^a^
NRS 6 h	6 (5.25–8)	5.5 (4–6)	<0.001^a^
NRS 12 h	6 (4–7)	4 (2–4)	<0.001^a^
NRS 18 h	6 (5–6.75)	2 (2–4)	<0.001^a^
NRS 24 h	5 (3.25–6)	2 (1–2)	<0.001^a^

^a^ Mann-Whitney-U-test.

Results are presented as median (interquartile range (IQR)); AUC, area under the curve; NRS, numeric rating scale.

Five patients in the control group required 2 mL of nalbuphine as rescue analgesia, while none in the pentoxifylline group required rescue analgesia. The incidence of post-operative complications was significantly higher in the control group (*P*= 0.01). However, the complication types were comparable between the two groups, as illustrated in Table 3.

**TABLE 3:** Secondary efficacy endpoints.

Parameter	Control group (n = 42)	Pentoxifylline group (n = 46)	*P*-value
Patients needed rescue analgesia, n (%)	5 (11.6)	0 (0)	0.02^b^
Complication incidence, n (%)	8 (19.0)	1 (2.2)	0.01^b^
Type of complication, n (%)			
Infection	2 (4.8)	0 (0.0)	0.06^b^
Seroma	3 (7.1)	1 (2.2)	
Wound dehiscence	3 (7.1)	0 (0.0)	
Time for suture removal (days), mean ± SD	19.3 ± 6.7	15.0 ± 4.4	0.001^a^

^a^ Student’s t-test.

^b^ Pearson Chi square.

**FIGURE 3:**
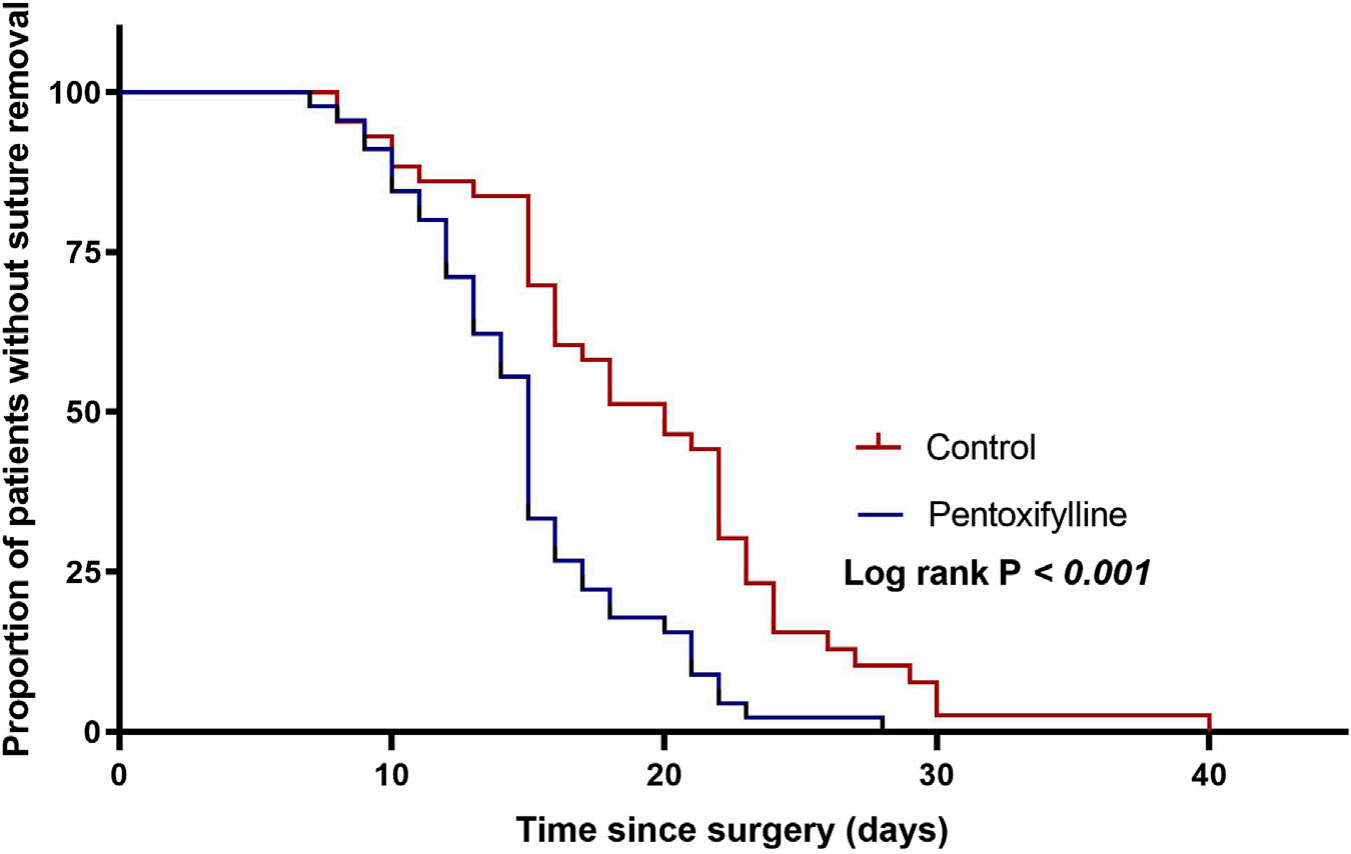
Kaplan-Meier survival curve representing the time to suture removal in the two groups.

Patients in the pentoxifylline group removed sutures earlier than those in the control group (mean ± SD: 15 ± 4.4 days vs. 19.3 ± 6.7 days, respectively; *P*= 0.001, Table 3). Figure 3 shows Kaplan-Meier survival estimates of suture removal in the two groups, with significant superiority in the pentoxifylline group (P-value of Log-rank test <0.001).

Table 4 compares the adverse effects experienced during the study in the control group and the pentoxifylline group. These results indicate that headache, dizziness, and nausea were among the most experienced side effects in the study with similar prevalence between the control group and the pentoxifylline group. Headache was observed in 23.9% of patients in the pentoxifylline group versus 40.5% in the control group (*P*= 0.10). The incidence of nausea was 30.4% in the pentoxifylline group compared to 14.3% in the control group (*P*= 0.07).

**TABLE 4:** Comparison of adverse effects between control and pentoxifylline groups.

Parameter	Control group (n = 42)	Pentoxifylline group (n = 46)	*P*-value
Nausea	6 (14.3)	14 (30.4)	0.07^a^
Vomiting	3 (7.1)	6 (13.0)	0.36^a^
Bloating	1 (2.4)	3 (6.5)	0.35^a^
Abdominal discomfort	2 (4.8)	3 (6.5)	0.72^a^
Dizziness	16 (38.1)	10 (21.7)	0.09^a^
Headache	17 (40.5)	11 (23.9)	0.10^a^
Constipation	1 (2.4)	0 (0)	0.29^a^

^a^ Pearson Chi square.

Results are described as n (%).

## 4 Discussion

This study represents the first randomized controlled trial to evaluate the efficacy of adjunctive pentoxifylline in alleviating acute pain in patients undergoing mastectomy. The primary outcome was postoperative pain scores, expressed as the cumulative AUC of the NRS scores, calculated using the trapezoidal rule. NRS scores were assessed every 6 h for 24 h after surgery to provide an integrated measure of pain levels. This approach has been utilized in various clinical trials to evaluate different outcomes, including pain (Lang-Illievich et al., 2020; Schwartz et al., 2024; Zhou et al., 2023). The current study demonstrated a statistically significant reduction in NRS scores for pain severity in the pentoxifylline group compared to the control group at 0, 6, 12, 18, 24 h post-operation. Previous studies have suggested that a reduction of 1.41 points in the NRS can be considered clinically significant. Thus, the difference in pain reduction between pentoxifylline group and control group was clinically important at all time points except for at 6 h post-operation (Kendrick and Strout, 2004).

The current study demonstrated a significant reduction in the cumulative AUC of the NRS scores for pain in the pentoxifylline group compared to the control group. Specifically, the median (IQR) AUC-NRS over 24 h was 153 (123–168) in the control group, compared to 90 (73.5–102) in the pentoxifylline group, representing a 41.17% reduction in the pentoxifylline group. This reduction in pain intensity, as measured by the AUC-NRS, was considered statistically significant (P < 0.001). Previous studies have suggested that a reduction of 33% in the overall pain intensity difference can be considered clinically significant (Farrar et al., 2003). The 41.17% reduction observed in the current study exceeds this threshold, emphasizing that the pain relief provided by pentoxifylline can be considered clinically important.

Furthermore, the present study found a significant difference in the required rescue analgesia between the two groups (*P*= 0.02), with the pentoxifylline group requiring less rescue analgesia compared to the control group. This further supports the analgesic efficacy of pentoxifylline in managing acute postsurgical pain.

Pentoxifylline perhaps exerts its analgesic effects through various mechanisms that collectively contribute to the reduction of acute pain. One significant pathway involves the inhibition of phosphodiesterase enzymes by pentoxifylline. This inhibition prompts an elevation in intracellular cyclic adenosine monophosphate (cAMP) levels. The increased cAMP levels subsequently activate protein kinase A (PKA), triggering a range of downstream effects relevant to pain and inflammation (Salehi et al., 2017). Firstly, by suppressing the translocation of nuclear factor-κB (NF-κB), pentoxifylline diminishes the transcription of pro-inflammatory cytokines like tumor necrosis factor-α, interleukin-1 (IL-1), and IL-6 (Ji et al., 2004; Ruan et al., 2021). This anti-inflammatory action may help mitigate the inflammatory response associated with pain. Secondly, pentoxifylline disrupts transforming growth factor-β1 signaling by activating the cAMP response element-binding protein. This disruption leads to a reduction in the production of pro-fibrotic molecules such as collagen, fibronectin, and α-smooth muscle actin (Chen et al., 2017; Lyons and Brennan, 2017). These cAMP-mediated mechanisms decrease the pro-inflammatory mediators, oxidative stress, and fibrosis, all of which are factors contributing to the development of acute pain.

In accordance with our findings, Nazemi et al. investigated the analgesic effects of pentoxifylline in patients undergoing laparoscopic appendectomy for acute postoperative pain. Their study revealed that patients who received pentoxifylline preemptively before surgery, exhibited significantly lower pain scores, reduced opioid consumption, and a lower incidence of secondary hyperalgesia compared to those in the placebo group pain (Nazemi et al., 2021).

In contrast, Szczepanik et al. reported differing results in patients undergoing cholecystectomy, where the administration of pentoxifylline immediately after anesthesia did not effectively alleviate postoperative pain (Szczepanik et al., 2004). This disparity in outcomes could be attributed to variations in the administration regimen and timing of pentoxifylline. In our study, pentoxifylline was administered orally as a preemptive measure, whereas in Szczepanik study it was administered intravenously immediately following anesthesia termination. Furthermore, discrepancies in the patient populations studied could also explain the differing results. Laparoscopic cholecystectomy, commonly associated with referred shoulder pain, may present a distinct pain profile. The shoulder pain often experienced results from irritation of the diaphragm during the laparoscopic procedure (Park, 2020).

Our study demonstrated a notable decrease in post-surgical complications with pentoxifylline compared to the control group (*P= 0.01*). Despite this discrepancy in incidence, the types of complications observed were similar in both study arms. Also, the pentoxifylline group exhibited a significant advancement in wound healing, evidenced by the early removal of sutures compared to the control group (P < 0.001). In line with these findings, pentoxifylline has shown positive outcomes in various wound healing contexts such as colorectal anastomosis, post-burn scars, radiation-induced injuries, and venous ulcers (Ahmadi and Khalili, 2016). Whether it was taken orally or parenterally, pentoxifylline presented favorable healing rates and reduced pain.

Proposed mechanisms underlying the beneficial effects of pentoxifylline on wound healing encompass its anti-inflammatory properties, enhancement of microcirculation and oxygen delivery, as well as its fibrinolytic characteristics (Ahmadi and Khalili, 2016). In line with these findings, preclinical investigations have demonstrated that systemic administration of pentoxifylline in mouse models leads to accelerated wound healing. Specifically, pentoxifylline has shown efficacy in improving the healing of colorectal anastomosis in animal studies and enhancing recovery from radiation-induced skin and soft tissue injuries (Ahmadi and Khalili, 2016). Clinically, research has highlighted the efficacy of pentoxifylline in promoting wound healing. For instance, a study revealed that systemic administration of pentoxifylline resulted in a remarkable 50% complete closure rate of lepromatous ulcers, *versus* 10% closure rate observed in the placebo group (Mikhael and El-Esawy, 2015). Additionally, pentoxifylline has been found to enhance the healing of venous leg ulcers, with a meta-analysis indicating a 21% increase in ulcer healing rates compared to placebo (Jull et al., 2012).

However, there were conflicting findings from experimental studies, which could be explained by the small sample size, starting time post-operatively or the shorter duration of treatment (Ahmadi and Khalili, 2016).

In terms of safety, pentoxifylline was safe and well-tolerated in the present study. Some side effects, like headache and nausea, were reported more frequently in the pentoxifylline group, yet it did not reach statistical significance. These findings were consistent with previous reports (Jull et al., 2012; Jull et al., 2000; Salhiyyah et al., 2015).

This study is constrained by several limitations. First, the relatively small number of patients enrolled necessitates cautious interpretation of the results, suggesting that the study should be regarded as a pilot investigation. Second, the subjective nature of the NRS score introduces a notable limitation. Third, the definition of wound healing based on suture removal is indirect and may not fully capture the dynamics of tissue repair; future studies could benefit from more direct measures such as structured dehiscence scoring systems. Fourth, the single-blinded study design and the lack of a placebo-controlled approach represent a significant limitation due to their potential impact on pain outcomes and may affect the reliability of self-reported outcomes. Finally, focusing on pain assessment only within the first 24 h postoperatively excludes evaluation of chronic pain. Further research with larger sample sizes, objective pain assessment tools, and extended follow-up periods is warranted to elucidate the impact of pentoxifylline in this patient population.

In conclusion, our findings suggest that preoperative oral administration of pentoxifylline in patients undergoing breast surgery may reduce postoperative pain in those patients. However, further trials are imperative to validate these findings.

## Data Availability

The raw data supporting the conclusions of this article will be made available by the authors, without undue reservation.
